# 

**Published:** 1934

**Authors:** 


### CONTENTS.

| | Page |
|---|---|
| The Twenty-Third Long Fox Memorial Lecture: Some Aspects of Heart Disease in Childhood. By F. J. Poynton, M.D., F.R.C.P. | 205 |
| Abscess of the Brain. By E. Watson-Williams, M.C., Ch.M., F.R.C.S. | 231 |
| Cerebral Dermoid. By P. Phillips, M.D., M.Sc., and D. M. Stone, M.D., D.P.H. | 247 |
| Reform of the Medical Curriculum. By A Medical Student | 253 |
| Reviews of Books | 2[illegible] |
| Editorial Notes | 2[illegible] |
| Obituaries: Vincent Middleton Coates, M.C., M.D., M.R.C.P. | 281 |
| Mary Shortridge Mules, L.M.S.S.A. | 282 |
| Meetings of Societies | 283 |
| The Medical Library of the University of Bristol | 286 |
| Publications Received | 288 |
| Local Medical Notes | 289 |

